# Nigrostriatal imaging in patients with clinically uncertain Parkinsonian syndromes: a head-to-head comparison between SPECT, PET, and MRI

**DOI:** 10.1007/s00259-026-08184-8

**Published:** 2026-09-17

**Authors:** Gerda Thomsen, Kristoffer Brendstrup-Brix, Claus Svarer, Ulrich Lindberg, Lars H. Pinborg, Karen Kettless, Ian Law, Steen G. Hasselbalch, Bo Biering-Sørensen, Gitte M. Knudsen

**Affiliations:** 1 Neurobiology Research Unit, Department of Neurology, Rigshospitalet, Copenhagen University Hospital, 6-8 Inge Lehmanns Vej, Copenhagen, DK-2100 Denmark; 2 Department of Clinical Medicine, Faculty of Health and Medical Sciences, University of Copenhagen, Copenhagen, Denmark; 3 Department of Clinical Physiology and Nuclear Medicine, Rigshospitalet, Copenhagen University Hospital, Copenhagen, Denmark; 4 Epilepsy Clinic, Department of Neurology, Rigshospitalet, Copenhagen University Hospital, Copenhagen, Denmark; 5 Siemens Healthcare A/S, Ballerup, Denmark; 6 Danish Dementia Research Centre, Department of Neurology, Rigshospitalet, Copenhagen University Hospital, Copenhagen, Denmark; 7 Movement Disorder and Pain Research Center, Department of Neurology, Rigshospitalet, Copenhagen University Hospital, Copenhagen, Denmark

**Keywords:** Parkinson’s disease, [^123^I]FP-CIT SPECT-CT, [^18^F]FE-PE2I PET-CT, Neuromelanin MRI, Iron-sensitive MRI

## Abstract

**Purpose:**

Neurodegenerative parkinsonism is characterized by degeneration of the dopaminergic nigrostriatal pathways and a reduction in nigral neuromelanin. Dopamine transporter (DAT) imaging assesses presynaptic striatal dopaminergic function and is typically performed with [^123^I]FP-CIT SPECT-CT, but has recently also become available with [^18^F]FE-PE2I PET-CT. Magnetic resonance imaging (MRI) can explore disease-related changes by quantifying neuromelanin and iron deposition associated with the loss of nigral neurons.

**Methods:**

In this study, we conducted a head-to-head comparison of [¹²³I]FP-CIT SPECT, [¹⁸F]FE-PE2I PET, and nigral MRI in 35 patients referred for diagnostic DAT imaging. Nigral MRI was performed with neuromelanin-sensitive gradient echo magnetization-transfer contrast MRI and iron-sensitive STrategically Acquired Gradient Echo (STAGE) MRI. Patient satisfaction with SPECT and PET was recorded via questionnaires.

**Results:**

Eighteen patients were diagnosed with neurodegenerative parkinsonism with a median symptom duration of 29 months. DAT availability as measured with SPECT and PET showed complete concordance across cases, correlated strongly, and demonstrated equally excellent diagnostic performance. Patient questionnaire items were either not different between modalities or in favor of SPECT. Nigral neuromelanin content was reduced in patients with neurodegenerative parkinsonism and provided intermediate diagnostic accuracy, with indications of lower neuromelanin with disease duration. Midbrain iron content did not differ between groups and provided no diagnostic information.

**Conclusion:**

Our findings indicate that in early-stage parkinsonism, DAT neuroimaging with SPECT and PET provides comparable and superior diagnostic value relative to nigral MRI.

**Supplementary Information:**

The online version contains supplementary material available at https://doi.org/10.1007/s00259-026-08184-8.

## Introduction

Parkinson’s disease (PD) is the second most common neurodegenerative disease [1] and the neurological disease with the fastest growing prevalence and disability world-wide [2, 3]. PD is characterized by widespread accumulation of intracellular aggregates of ɑ-synuclein [4] and neuronal cell loss, which in the substantia nigra results in dopaminergic denervation of the caudate nucleus and putamen [5]. The clinical hallmarks of PD are motor symptoms resulting from the nigrostriatal neurodegeneration and include bradykinesia, rigidity, and tremor [6]. These symptoms are, however, also seen in other neurodegenerative diseases and in conditions of vascular, toxic, infectious, or autoimmune etiology [7]. Diagnosing PD therefore pose a great challenge to clinicians and may cause delays in diagnosis and optimal treatment [7–11].

For decades, imaging of the striatal dopamine transporter (DAT) has supported the diagnostic assessment of parkinsonian syndromes, with reduced striatal DAT binding seen in PD and other neurodegenerative parkinsonian disorders [12]. A gold standard diagnostic examination for patients with parkinsonism of uncertain etiology has been single-photon emission computer tomography (SPECT) with [^123^I]N-ω-fluoropropyl-2β-carbo-methoxy-3β-(4-iodophenyl)nortropane ([^123^I]FP-CIT) [13]. More recently, positron emission tomography (PET) with the radioligand [^18^F](E)-N-(3-iodo-prop-2-enyl)-2β-carbofluoroethoxy-3β-(4’-methyl-phenyl)nortropane ([^18^F]FE-PE2I) has emerged as an alternative for DAT imaging [14]. [^18^F]FE-PE2I PET has superior image resolution and shorter waiting times from tracer injection to scanning compared to conventional [^123^I]FP-CIT SPECT [15, 16]. However, recent technical advances in SPECT systems, specifically the introduction of high-resolution multi-pinhole collimators (MPH), may bring the image resolution and richness of detail of SPECT imaging up to par with that of PET [17, 18]. While previous studies have examined the diagnostic accuracy of [^18^F]FE-PE2I PET relative to conventional SPECT with [^123^I]FP-CIT, the outcome of a head-to-head comparison between a high-resolution MPH collimator SPECT system and PET remains to be unraveled.

Beyond nuclear medicine, other emerging imaging techniques for assessing nigrostriatal integrity include magnetic resonance imaging (MRI) methods such as neuromelanin (NM) MRI and iron-sensitive MRI. These new imaging techniques offer significant potential to enhance diagnostic accuracy in the evaluation of patients presenting with parkinsonian symptoms [19–21]. NM is a chelator of iron, accumulates with age in dopaminergic neurons in the substantia nigra, and is detectable with MRI [20, 22]. Neurodegeneration of the substantia nigra leads to reductions in NM-MRI signal [22, 23] and increases in iron content, which due to its paramagnetic properties is detectable with susceptibility-based MRI techniques. NM- and iron-sensitive MRI may therefore provide fast and radiation-free methods for assessing nigrostriatal function. While NM-MRI and quantitative susceptibility maps (QSM) have been examined in patients with PD [21, 24], their direct comparison with DAT imaging remains to be investigated.

Here, we perform a head-to-head comparison between [¹²³I]FP-CIT SPECT, [¹⁸F]FE-PE2I PET, and NM- and iron-sensitive MRI in a prospectively recruited cohort of patients with clinically uncertain parkinsonian syndromes. The study also incorporates patient-reported questionnaires with a particular focus on patient experience related to comfort during examination and waiting time from injection to scanning.

## Materials and methods

### Subjects

Thirty-five patients referred for diagnostic DAT neuroimaging were included from Oct. 2021 to Jan. 2024 (27 males, mean age [IQR] = 72.0 [65.6–77]; Table 1). Fifteen healthy controls (HC; seven males, mean age [IQR] = 64.0 [57.35–71.5]) were recruited from an in-house database. Patients underwent imaging with [^123^I]FP-CIT SPECT/CT, [^18^F]FE-PE2I PET/CT, and MRI, whereas HCs only underwent MRI. In three patients and five HCs, MRI was aborted due to claustrophobia or scanner malfunction. Final diagnoses were determined by neurologists specialized in movement disorders and extracted in Sep. 2025 (median follow-up time [IQR] = 2.6 [2.3–3.8] years). Diagnoses were based jointly on all available information of clinical relevance including clinical examinations, DAT imaging, and structural neuroimaging.

**Table 1:** Cohort characteristics. Hoehn-Yahr stage and duration of symptoms only provided for patients with PD and/or LBD

	Patients *n* = 35	Healthy controls *n* = 15
Age (median [IQR])	72.0 [65.6–77]	64.0 [57.35–71.5]
Sex (% female [female/male])	22.9 [8/27]	53.3 [8/7]
Referral diagnosis (% [n])	100 [35]	-
PD	68.6 [24]	-
APD	14.3 [5]	-
iNPH	8.6 [3]	-
LBD	5.7 [2]	-
Tremor	2.9 [1]	-
Final diagnosis (% [n])	100 [35]	-
PD	37.1 [13]	-
Vascular pseudoparkinsonism	11.4 [4]	-
Drug-induced parkinsonism	8.6 [3]	-
iNPH	8.6 [3]	-
PDD/LBD	8.6 [3]	-
None	8.6 [3]	-
Tremor	5.7 [2]	-
MCI	5.7 [2]	-
PSP	2.9 [1]	-
Non-specific neurodegenerative basal ganglia disease	2.9 [1]	-
Hoehn-Yahr stage (mean [range])	1.86 [1–3]	-
Duration of symptoms (median [IQR])	29 [12–57]	-

Three patients and five healthy controls did not complete MRI due to claustrophobia or scanner malfunction. PD, Parkinson’s disease; APD, atypical Parkinson’s disease (i.e., progressive supranuclear palsy [PSP], multiple system atrophy, corticobasal degeneration); iNPH, idiopathic normal pressure hydrocephalus; PDD, Parkinson’s disease dementia; LBD, Lewy body dementia; MCI, mild cognitive impairment

DAT imaging with PET and SPECT were acquired on separate days (on average 18 days apart [range: 1 to 105 days]; DAT SPECT was performed first in 19 patients). MRI was performed on the day of DAT SPECT imaging.

Inclusion criteria were age between 30 and 90 years, absence of severe somatic disorders or contraindications for receiving radioactive tracers and/or sodium perchlorate blockade. Exclusion criteria were pregnancy or lactation and contraindication to MRI. Healthy controls were also excluded in case of a Mini-Mental State Examination score < 27, daily intake of antipsychotics or other medicines known to affect the dopamine system, severe hypertension (> 200 mmHg systolic or > 115 mmHg diastolic pressure), weekly alcohol consumption > 14 units for women and > 21 units for men, regular use of illicit substances including cannabinoids within the past three months (based on participant reports), or recent (< 1 year) exposure to significant radioactivity (> 10 mSv). Eventual medication did not change during the trial period.

All patients and HCs were thoroughly informed about the study and gave informed written consent. The study was performed in accordance with the ethical standards of the Declarations of Helsinki and was approved by the ethical committee of Copenhagen Capital Region (protocol number: H-19082644). All data was handled according to regulations by The Danish Data Protection Agency.

### SPECT

#### Tracer injection and imaging procedures

To block thyroidal uptake of free radioiodine, 200 mg of sodium perchlorate was administered intravenously 5–10 min. before tracer injection. SPECT brain imaging with DaTscan™ (GE Healthcare B.V, Eindhoven, the Netherlands) was performed three hours after injection of [^123^I]FP-CIT (^123^I-ioflupane; mean effective dose [range] = 195 MBq [182–205] MBq). Three consecutive 10 min. SPECT scans each covering 360 degrees of rotation were performed, yielding a total scan time of 30 min. A three-headed brain SPECT-CT camera (AnyScan Trio SC, Mediso Medical Imaging Systems, Budapest, Hungary) with general purpose brain MPH collimators was used for DAT-scanning with a 20% width energy window centered at 159 keV. A localizer CT scan was used for patient positioning and a low dose CT was performed for attenuation correction. All original images were reconstructed in Mediso software (using the Tera-Tomo Q procedure) for 10, 20, and 30 min. scan durations by adding images for the multiple acquired frames. 2D projection (raw) data was acquired in frame mode with three heads at 24 angles yielding 72 angles in total with an isotropic voxel size of 2.3 mm^3^. The projection data was reconstructed with an iterative ordered subsets expectation algorithm (36 iterations and three subsets) including CT-based attenuation correction and scatter correction with a Monte Carlo algorithm (medium quality setting). The resulting isotropic voxel size of the reconstructed images was 1.8 mm^3^.

#### ROI delineation and SBR calculation

As a quantitative measure of tracer uptake, we used the ratio of specifically bound radioligand to that of non-displaceable radioligand in tissue. The specific binding ratio (SBR) was defined as

1 $$\begin{array}{c}SBR=\frac{C-{C}_{ref}}{{C}_{ref}}\end{array}$$

where *c* is the activity concentration in the putamen, caudate nucleus, or whole striatum, and *c*_*ref*_ the concentration in the reference region, in this case the occipital lobe [25].

Regions of interest (ROIs) were delineated semi-automatically using a template-based approach (*DATquan*) [25]. Asymmetry indices (AI) were calculated in the striatum (SAI), putamen, and caudate nucleus as follows:

2 $$\begin{array}{c}AI=\left|\frac{SB{R}_{rh}\:-\:SB{R}_{lh}}{{SBR}_{rh}\:+\:SB{R}_{lh}}\right|\end{array}$$

where *SBR*_*rh*_ is the SBR value in the right hemisphere and *SBR*_*lh*_ is the SBR value in the left hemisphere.

SPECT images were evaluated clinically and categorized as normal or abnormal based on visual assessments in combination with comparisons of SBR values to an in-house reference database of healthy controls.

### PET

#### Tracer injection and imaging procedures

Patients were PET scanned using a Siemens PET/CT scanner (Siemens Healthineers, Forchheim, Germany) using the models Biograph Vision (*n* = 9) and mCT (*n* = 26). Scans were performed as a static 10 min. frame 30 min. after the injection of 200 MBq [^18^F]FE-PE2I [195–220 MBq]. The median activity concentration of [^18^F]FE-PE2I at end synthesis was 450 MBq/mL. PET reconstruction was performed by 3D OP-OSEM + TOF and a 4 mm gaussian filter with either 12 iterations, 5 subsets (0.83 × 0.83 × 1.65 mm^3^ voxel size) or 4 iterations, 21 subsets (0.68 × 0.68 × 2.03 mm^3^ voxel size), respectively for the two scanners. A low-dose CT scan performed directly before PET was used for attenuation correction.

#### ROI delineation and SBR calculation

Semi-quantitative PET SBR values were obtained for the striatum, caudate nucleus, and putamen using an in-house, automated segmentation tool described in detail elsewhere [16]. The cerebellar cortex was chosen as a reference region [26]. SAIs, putamen, and caudate nucleus asymmetry indices were calculated similar to that of SPECT (Eq. 2). PET images were evaluated clinically based on both visual and semi-quantitative analysis.

### MRI

#### Acquisition parameters

MRI was acquired on a 3T MAGNETOM Siemens Prisma system (Siemens Healthineers, Erlangen, Germany) with a 64-channel head and neck coil. In four patients and one healthy control, the diameter of the 64-channel head coil was too small, and MRI was instead acquired with a larger diameter 20-channel head and neck coil. Images acquired with the 20-channel head and neck coil did not differ from those acquired with the 64-channel coil in terms of image quality or in the computed outcome variables.

NM-MRI was acquired with a 3D multi-echo gradient echo sequence with a magnetization transfer contrast (MTC) pulse. Quantitative data was acquired with two sequential flow compensated bipolar 3D gradient echo sequences with identical imaging parameters at low and high flip angle (6° and 27°). Sequence specifications are listed in the supplementary information.

#### STAGE reconstruction

STAGE data was analyzed using a research version of the STAGE calculation software (Version 3.2.5, SpinTech MRI, Bingham Farms, United States). Quantitative susceptibility measures were calculated using multi echo iterative susceptibility weighted imaging maps (iSWIM). The reported QSM values were extracted from the iSWIM generated by the software.

#### Preprocessing

Briefly, NM-sensitive images were corrected for intensity non-uniformity with ANTs’ *N4BiasFieldCorrection* algorithm [27]. NM-sensitive images and QSM were then median filtered using MATLAB’s *medfilt3* (MATLAB version: 25.1.0.2943329 [R2025a], Natick, Massachusetts: The MathWorks Inc.; 2025). Further preprocessing details are available in the supplementary information.

ROIs on NM-sensitive images and QSM were delineated in MATLAB using in-house software. All analyses were performed by one reader blinded to clinical status and subject identifiers. ROI delineation was performed on two separate occasions separated by one week, and only voxels included on both occasions were used for analysis.

Bilateral reference regions were placed in the anterolateral cerebral crus and used for z-score normalization of signal intensities. For each slice, ROI boundaries were then defined by isocontours determined from signal intensities surrounding a manually defined ROI center (see further details in the supplementary information). The sum of z-scores inside the ROI was calculated and used as outcome measure since it reflects both the intensity and volume of the ROI. The strategy of using z-score normalized maps instead of raw signal intensities was employed in order to improve observer reproducibility and reduce noise, since voxels with only background signal on average will add up to a z-score of zero and thus not contribute to the sum. These outcome measures are for the remainder of the manuscript referred to as substantia nigra NM and midbrain iron content, respectively. Exemplary ROIs are presented in Fig. S1 and S2.

One HC had values of midbrain iron content exceeding the group-wise third quantile by approximately three times the IQR and was thus removed from analyses on iron content.

### Patient-reported experience measures

After SPECT and PET scans, patients were asked to complete a questionnaire assessing comfort and satisfaction with the imaging procedures. The questionnaire covered pre-scan procedures (including waiting time from tracer injection to scanning), scanning procedures (relating to comfort, quality of care, and interaction with clinical staff), and overall satisfaction. Responses were recorded using a five-point Likert scale, with a score of one indicating the lowest and a score of five indicating the highest level of satisfaction. Analysis was completed on the following items: Waiting time from tracer injection to scan, tracer injection, the scanning procedure, and overall experience.

### Statistics

#### Patient-reported experience measures

Differences in patient-reported comfort and satisfaction between PET and SPECT imaging were tested with unpaired, two-tailed t-tests. P-values were corrected for multiple comparisons with the Benjamini-Hochberg method (four tests). Effect sizes were calculated as Cohen’s *d*.

#### Comparison between modalities

DAT SPECT and PET outcome measures were compared with Pearson correlations tests and Bland-Altman plots with limits of agreement at the mean difference ± 1.96 standard deviations. A one-sample, two-tailed t-test was performed on the differences in order to test for systematic bias in either direction. P-values are reported without correction for multiple comparisons. Substantia nigra NM was tested against SPECT and PET outcome measures with Pearson correlation tests.

Receiver operating characteristics (ROC) curves for all imaging outcome measures were computed with the plotROC R package [28].

#### Observer reproducibility in ROI delineation on NM-MRI and QSM

NM and iron content from two repetitions of ROI delineations were tested against each other with Pearson correlation tests and Bland-Altman plots. One-sample, two-tailed t-tests were used for testing systematic bias. P-values are reported without correction.

#### Group differences in substantia nigra NM and midbrain iron content

A linear regression model was fitted to substantia nigra NM and midbrain iron contents from HCs only and used for subsequent adjustment for age and intracranial volume in both healthy controls and patients (Fig. S3). Effect sizes between diagnostic groups were calculated as Cohen’s *d* and differences were tested with unpaired, two-tailed t-tests. P-values were corrected for multiple comparisons with the Benjamini-Hochberg method (three tests per group).

### Data sharing

The dataset supporting the conclusions of this article can be made available upon reasonable request, as permitted by applicable law.

## Results

Referral and final diagnoses are listed in Table 1. Patients were referred on suspicion of five different diagnoses of which the most common were PD (*n* = 24 [68.6%]) and atypical PD (APD; *n* = 5 [14.3%]). Eighteen patients were diagnosed with neurodegenerative parkinsonism, of which 13 had PD, three had PD with dementia or Lewy body dementia (PDD/LBD), one had progressive supranuclear palsy, and one had non-specific neurodegenerative basal ganglia disease. Mean Hoehn and Yahr stage for the PD patients was 1.86 (range = 1 to 3) with a median symptom duration at the time of DAT imaging of 29.0 months (IQR = 12–57; Table 1).

**Fig. 1:**
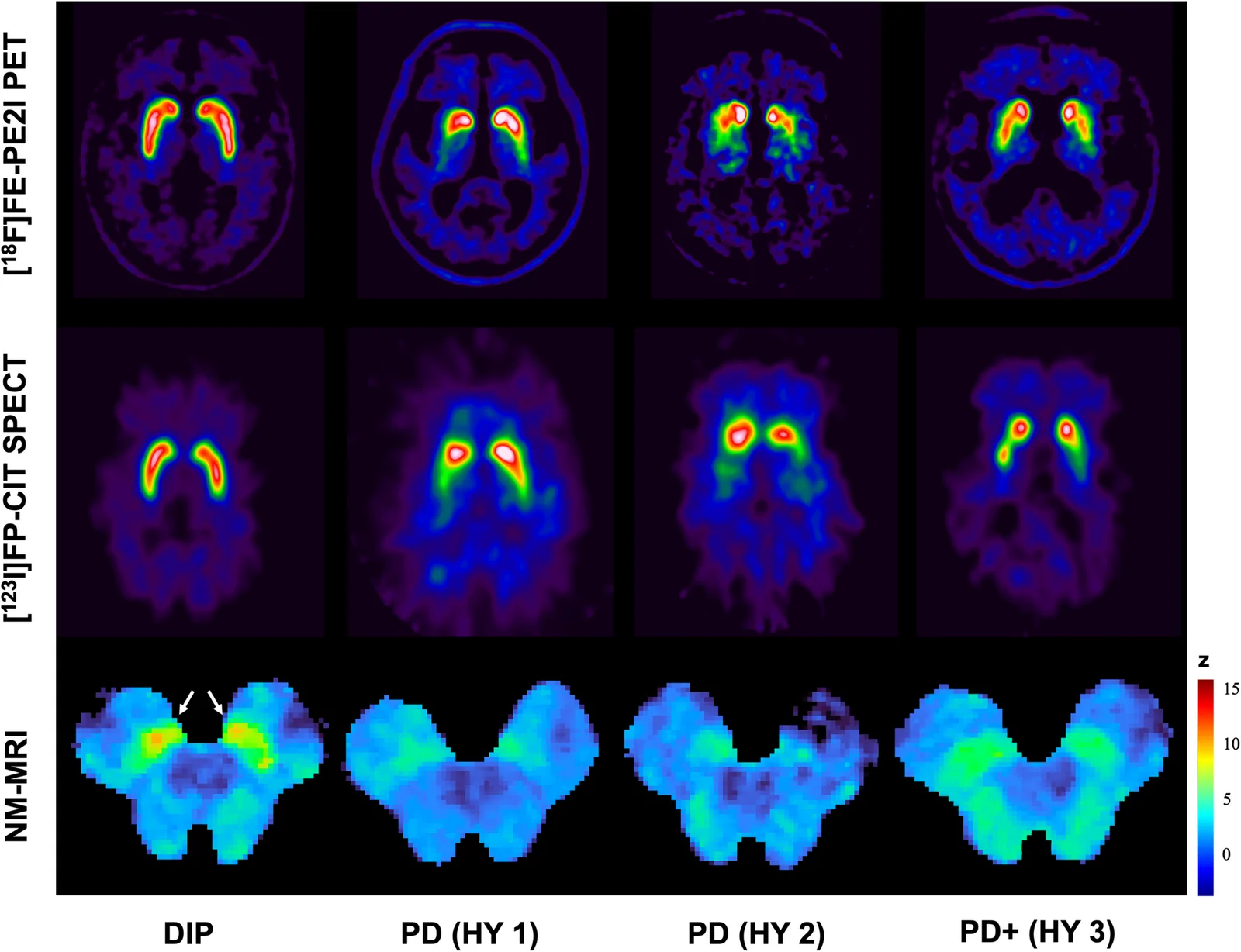
Exemplary [^18^F]FE-PE2I PET, [^123^I]FP-CIT SPECT, and neuromelanin-sensitive MR (NM-MRI) images for three patients with PD and one patient with drug-induced parkinsonism (DIP). Visual assessment of PET and SPECT images reveal normal uptake in the caudate nucleus and putamen for the patient with DIP and uni- or bilaterally reduced uptake for all PD patients. Visual evaluation of z-score maps derived from NM-MRI reveal substantia nigra hyperintensity in the patient with DIP (white arrows) and varying degrees of relative hypointensity in the patients with PD. Note the apparent substantia nigra NM hyperintensity in the patient with PD+

Representative SPECT, PET, and NM-MRI from four patients are shown in Fig. 1. DAT scans were normal on both SPECT and PET in 17 patients (48.6%) and abnormal on both modalities in 18 patients (51.4%). One patient demonstrated normal DAT uptake on PET but slightly reduced uptake on SPECT. However, based on clinical evaluation incorporating visual assessment and SBR values, this SPECT scan was classified as normal.

### Patient satisfaction and scanner comfort

Twenty-seven patients completed questionnaires on satisfaction with SPECT and PET imaging procedures (Table S1). Patients reported higher satisfaction with SPECT imaging with regards to the general scanning experience (Cohen’s *d* [95% CI] = 0.59 [0.18, 1.00], *p*_*FWER*_ = 0.02). No statistically significant differences were found in relation to waiting time from injection to scan, injection of tracer, and the overall scanning procedure (all *p*_*FWER*_ > 0.27).

### Head-to-head comparison between DAT SPECT and PET

**Fig. 2:**
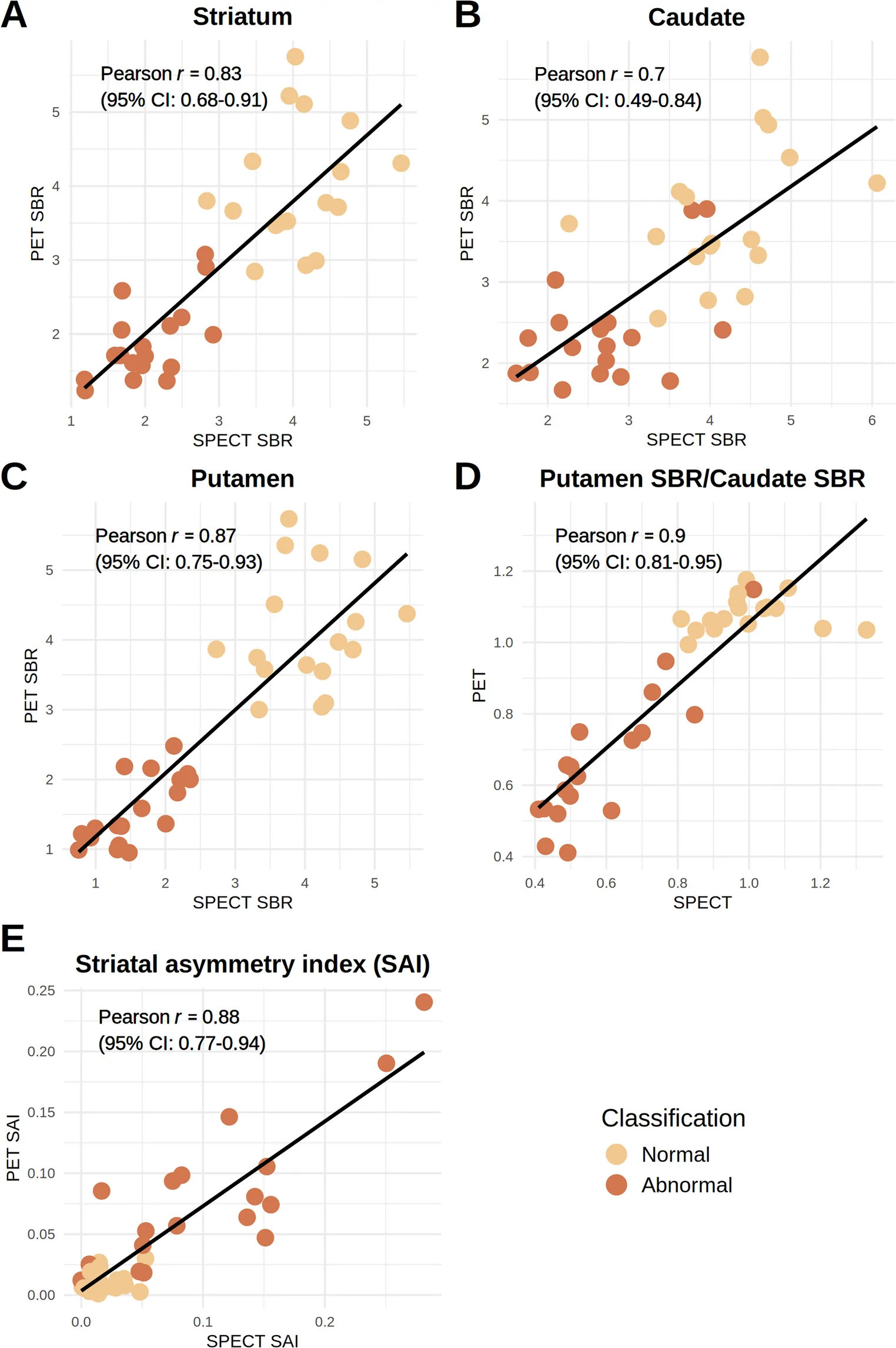
Correlations between specific binding ratios (SBR) and related quantitative measures from [^18^F]FE-PE2I PET and [^123^I]FP-CIT SPECT. Classification defines the clinical evaluation based on visual inspection and DAT SBR values. **A**,** B**,** C** Correlation between PET SBR and SPECT SBR in the striatum, caudate nucleus, and putamen. **D** Correlation between the relation between putamen and caudate nucleus SBR as evaluated on PET relative to SPECT images. **E** Correlation between striatal asymmetry index (SAI) as evaluated on SPECT relative to PET

Pearson correlation coefficients between quantitative outcome measures from SPECT and PET ranged from 0.7 to 0.9 (95% CIs ranging from 0.68 to 0.95; Fig. 2), with almost perfect correlation between SPECT and PET putamen/caudate nucleus SBR ratios (Pearson *r* [95% CI] = 0.9 [0.81, 0.95]; Fig. 2D). Similar results were found for putamen and caudate nucleus asymmetry indices (Fig. S4). For striatal, caudate nucleus, and putamen SBR values, limits of agreement were approximately ± 50% of the mean SBR values (Fig. S5A-C). Limits of agreement were smaller for putamen/caudate nucleus SBR ratios at approximately ± 25% of mean ratios, and larger for SAIs at around ± 120% of mean values (Fig. S5D-E). Relative to SPECT, PET values were lower for caudate nucleus SBR and SAI (Fig. S5B, S5E) and higher for putamen/caudate nucleus SBR ratios (Fig. S5D).

### Differences in NM and iron content between diagnostic groups

Substantia nigra NM content calculated on the first and second repetition of ROI delineation correlated with a Pearson correlation coefficient of 0.95 (95% CI: 0.91, 0.97; Fig. S1C). Similar results were achieved for midbrain iron content (Pearson correlation coefficient of 0.97 [95% CI: 0.94, 0.98]; Fig. S2C). Estimates of NM and iron content were higher on the second delineation; for NM, this bias increased with higher outcome values (Fig. S1D, S2D).

While substantia nigra NM content did not differ between patients with neurodegenerative parkinsonism and patients with other neurological disease (Cohen’s *d* [95% CI] = -0.56 [-1.51, 0.41], *p*_*FWER*_ = 0.33), patients with neurodegenerative parkinsonism had lower substantia nigra NM content compared to participants with no neurological disease, i.e., healthy controls and patients with no organic diagnosis (Cohen’s *d* [95% CI] = -1.03 [-1.75, -0.29], *p*_*FWER*_ = 0.01; Fig. S6B). Similarly, a trend was found in the direction of lower NM content in those with a DAT scan deemed abnormal on clinical evaluation (Cohen’s *d* [95% CI] = -0.83 [-1.58, -0.06], *p*_*FWER*_ = 0.07; Fig. S6C). Midbrain iron content was not different between patients with neurodegenerative parkinsonism relative to patients with other neurological disease (Cohen’s *d* [95% CI] = -0.18 [-1.13, 0.77], *p*_*FWER*_ = 0.89) or participants with no neurological disease (Cohen’s *d* [95% CI] = 0.16 [-0.54, 0.85], *p*_*FWER*_ = 0.89; Fig. S7B), nor was it different in those with an abnormal relative to a normal DAT scan (Cohen’s *d* [95% CI] = -0.05 [-0.78, 0.68], *p*_*FWER*_ = 0.89; Fig. S7C). A trend towards a negative association between symptom duration and NM content was found (Pearson’s *r* [95% CI] = -0.36 [-0.76, 0.23], *p* = 0.22; Fig. S8).

### Diagnostic performances and correlations between outcome measures

**Fig. 3:**
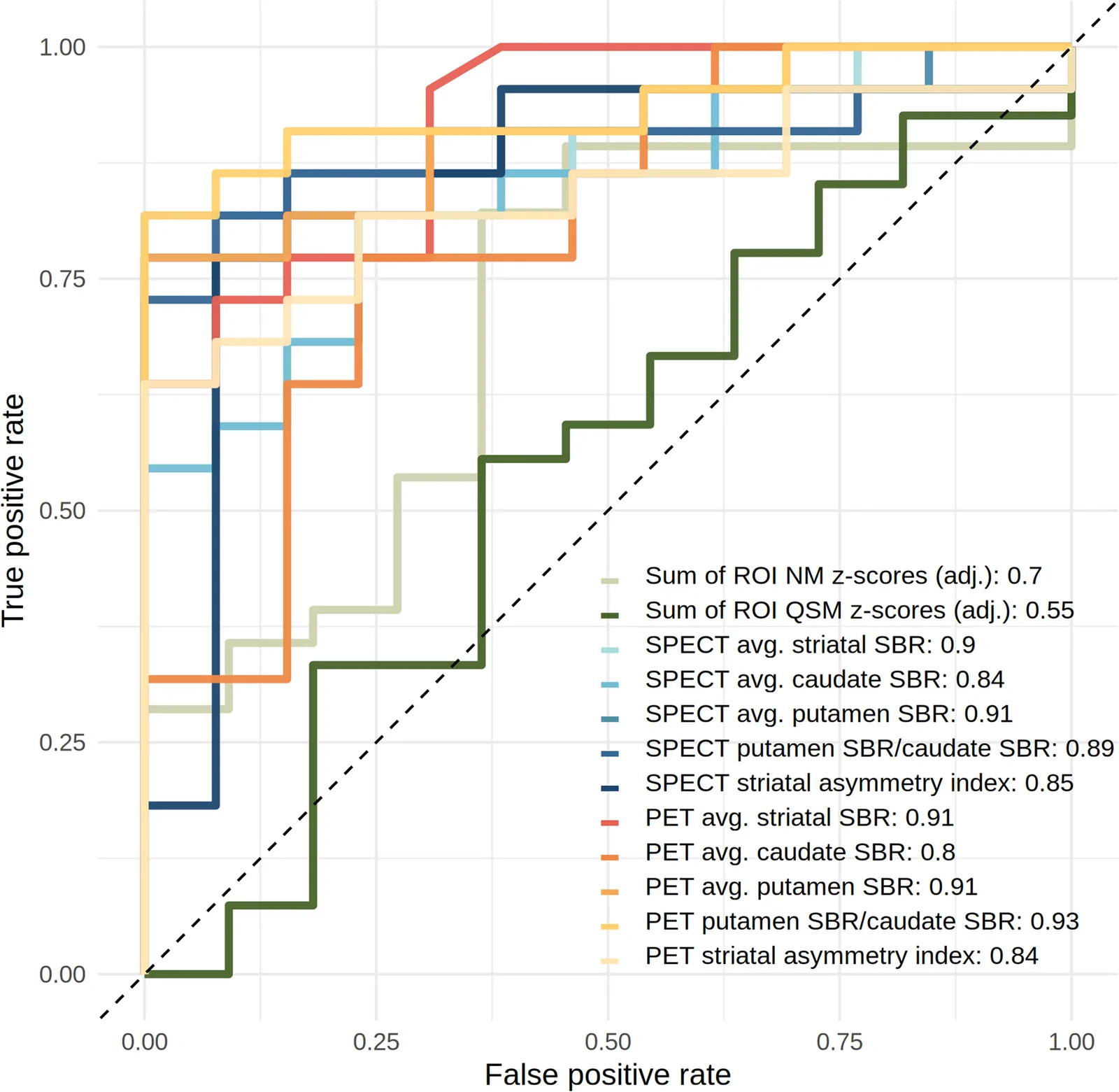
Receiver operating characteristics (ROC) curves. ROC curves for NM- and iron-sensitive MRI (green), SPECT (blue colors), and PET (orange and yellow colors) outcome measures. Areas under the curve (AUC) for each ROC curve are displayed under the diagonal

ROC curves revealed diagnostic performances of regional SBR values, putamen asymmetry indices, and SAIs in the range of AUC = 0.84 to 0.92 for SPECT and AUC = 0.80 to 0.93 for PET. Caudate nucleus asymmetry indices performed worse at AUC = 0.56 for SPECT and AUC = 0.71 for PET. Substantia nigra NM content performed at an intermediate level with an AUC of 0.7, while midbrain iron content performed at chance level at AUC = 0.55 (Fig. 3, Fig. S4).

**Fig. 4:**
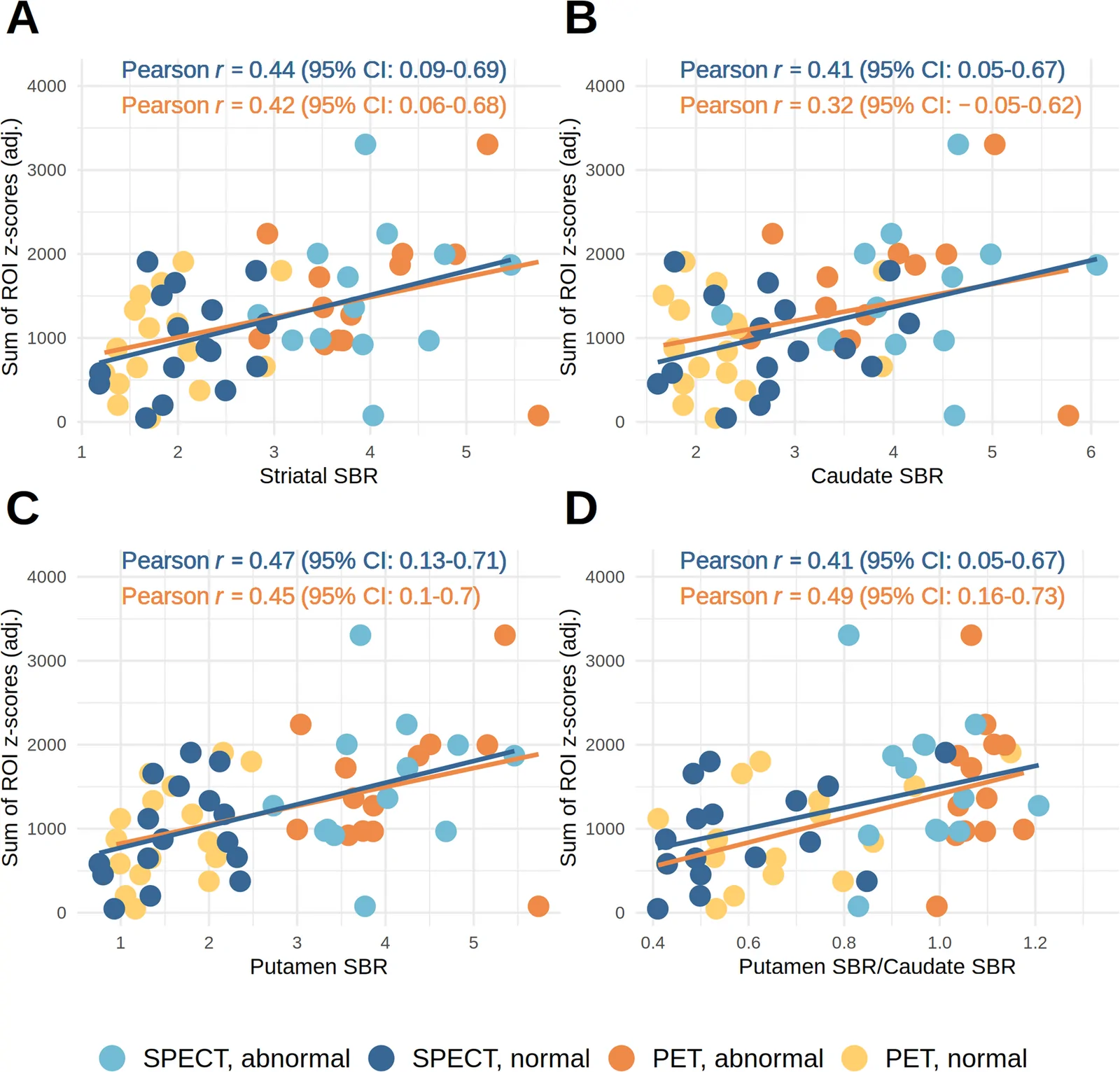
Correlations between standardized neuromelanin hyperintensities and DAT imaging outcome measures. Datapoints are colored according to modality (SPECT in blue colors and PET in orange and yellow) and classification (abnormal or normal) based on clinical evaluation. Pearson correlation tests were performed between neuromelanin MRI outcome measures and outcome measures from each of the two DAT imaging techniques (PET and SPECT). Striatal asymmetry indices were bimodally distributed and were therefore not included in correlation analyses.

Correlations between NM values and DAT imaging outcome measures were similar for PET and SPECT with Pearson coefficients ranging from 0.32 to 0.49 (95% CIs ranging from − 0.05 to 0.73; Fig. 4). Correlations were strongest between substantia nigra NM content and putamen/caudate nucleus SBR ratios for PET (Pearson’s *r* [95% CI] = 0.49 [0.16, 0.73]; Fig. 4D) and between substantia nigra NM content and putamen SBR values for SPECT (Pearson’s *r* [95% CI] = 0.47 [0.13, 0.71]; Fig. 4C).

## Discussion

State-of-the-art high-resolution SPECT-CT with [^123^I]FP-CIT provided images with the same high richness of detail as [^18^F]FE-PE2I PET, translating to a perfect agreement on diagnostic information and strong correlations between quantitative measures. Importantly, both methods yielded excellent discriminative properties in differentiating patients with neurodegenerative parkinsonism from healthy controls and patients with non-neurodegenerative parkinsonism (e.g. vascular pseudoparkinsonism or drug-induced parkinsonism; see Table 1). Aligning with previous studies on the spatiotemporal evolution of PD [29], measures including putamen DAT availability yielded the highest reproducibility and diagnostic accuracies across modalities. Patient comfort and satisfaction were not adversely affected by the longer injection-to-scan interval associated with SPECT imaging. On the contrary, overall satisfaction was higher for SPECT than for PET, likely reflecting increased attention to patient needs during the add-on SPECT examination.

DAT imaging with SPECT is typically performed with dual- or triple-head SPECT cameras with low-energy, high-resolution, parallel-hole collimators [30]. Here, we employed the AnyScan Trio SPECT-CT, a new three-headed SPECT camera with dedicated brain MPH collimators which have 2.4 times better sensitivity in the FoV (~ 250 cps/MBq at the edges of the FoV) compared to conventional SPECT systems and even higher sensitivity in the FoV center [31, 32]. This increase in image quality may together with a lower cost of scanner, comparable patient comfort, and greater scanner accessibility [33] (see Table 2 for a comparison of SPECT and PET) put SPECT ahead of PET for DAT imaging. Conversely, previous literature suggests lower specificity of conventional SPECT relative to PET for DAT imaging [34, 35]. DAT imaging with PET therefore provides a reliable and possibly more accurate alternative when on-site cyclotron and radiochemistry facilities are available or the tracer is with-in acceptable transport distance.

**Table 2:** Head-to-head comparison of clinical setups and workflow with DAT SPECT and DAT PET

	DAT SPECT	DAT PET
Isotope	[^123^I]FP-CIT	[^18^F]FE-PE2I
Half-life	13 h 13 min	1 h 50 min
Tracer production	No on-site cyclotron and radiochemistry required	Requires cyclotron and radiochemistry facilities on-site or within reasonable transport distance
Block thyroid ^123^I uptake	Yes	No
Scanner	AnyScan Trio SPECT-CT MPH collimator	PET-CT BiographTM RT, Siemens
Spatial resolution	6 mm^1^	5 mm
Time in scanner	20–30 min^2^	10 min
Time from injection to scanning	3 h	30 min
Radiation exposure	185 MBq/4.6 mSv	185 MBq/4.3 mSv
Semi-quantitative value	Yes (SBR)	Yes (SBR)
Sensitivity to SSRI treatment	Yes^3^	No^4*^
Cost of isotope	650 € incl. transport cost	Variable: 300–1200 €
Cost of scanner	750 000 €	2 000 000 €
Scanner staff time (incl. injection)	1.3 h	1 h

(1) Mathies F. EJNMMI Res. 2022. (2) Thomsen G. EJNMMI Phys. 2026. (3) Ziebell M. J. Nucl. Med. 2010. (4) Justesen TEH. EJNMMI Res. 2023. ^*^Sertraline shows affinity to DAT and therefore influences [¹⁸F]FE-PE2I imaging

For MRI-based outcome measures, we observed reduced NM content in the substantia nigra of patients with neurodegenerative parkinsonism compared with individuals without neurological disease. These group-wise differences did, however, only translate into moderate diagnostic accuracy at the individual subject level. In contrast, midbrain iron content showed no group-level differences and demonstrated diagnostic performance at chance level.

A trend toward lower substantia nigra NM content with increasing PD symptom duration suggests that the modest subject-level performance of NM measures may, at least in part, be attributable to the early disease stage of our cohort. Although substantia nigra neuronal loss is known to precede the onset of motor symptoms and substantia nigra NM therefore could be sensitive to PD pathology early in disease progression [36–38], recent studies are consistent with our findings in suggesting that striatal dopaminergic dysfunction precedes nigral NM loss and iron accumulation [29, 39]. Supporting this notion, meta-analyses of NM- and iron-sensitive MRI in PD report lower diagnostic accuracy in patients with disease durations shorter than five years compared with those with longer disease duration, estimating an overall AUC of approximately 0.84 for substantia nigra NM content in early PD [40, 41]. Given the even shorter median disease duration in our cohort, this likely explains the discrepancy with previous research [40, 42].

Further complicating comparisons across studies, a wide range of methods has been used to define substantia nigra regions of interest, including manual delineation and semi- or fully automated techniques [21, 40, 43]. Outcome measures have likewise varied from qualitative assessment of the “swallow-tail” sign to quantitative analyses of raw signal intensity or contrast-to-noise ratios. While meta-analyses suggest that substantia nigra volume may outperform signal intensity [40], both likely capture complementary aspects of nigral integrity [44]. In this study, we quantified NM by summing z-scores within a semi-automatically delineated NM-hyperintense region, thereby integrating information on signal intensity and volume while remaining relatively robust to noise and overestimation. We propose further evaluation of this approach, potentially in combination with automated ROI delineation methods [45–47]. Ultimately, standardized acquisition protocols, processing pipelines, and quantification strategies are required to validate nigral MRI as a reliable biomarker and facilitate its implementation in routine clinical practice [8].

Some limitations of our study should be acknowledged. As with any imaging study in PD, diagnoses were based on best clinical practice rather than neurohistological confirmation, which remains the definitive gold standard for synucleinopathies. Nevertheless, the reliability of clinical diagnoses in our cohort was high, supported by relatively long follow-up periods ranging from 20 to 48 months. Diagnostic uncertainty is an inherent challenge in real-world clinical practice [8], and our study design therefore reflects these conditions accurately. To enhance translatability, patients were consecutively recruited in an unselected manner, resulting in an even distribution of normal and abnormal scans. However, this inclusion strategy also led to a relatively small number of patients with APD, limiting our ability to draw specific conclusions for this subgroup compared with idiopathic PD. Larger, multicenter studies including greater numbers of patients with APD are needed to assess the generalizability of our findings.

In conclusion, in this heterogeneous cohort of patients with early-stage PD, APD, and non-neurodegenerative parkinsonism, DAT availability measured by SPECT and PET showed strong correlations and provided equally high diagnostic accuracy. NM content in the substantia nigra was reduced in patients with neurodegenerative parkinsonism compared with individuals without neurological disease, but at the individual level, NM served only as an intermediate diagnostic biomarker. Midbrain iron content did not differ across groups and offered no diagnostic utility. These results indicate that SPECT with MPH collimators and PET are equivalent for DAT imaging and outperform MRI-based biomarkers in early PD, suggesting that the choice of modality may be guided by local availability and implementation costs. Further studies are needed to optimize, standardize, and evaluate MRI-based biomarkers for clinical use.

## Supplementary Information

Below is the link to the electronic supplementary material.

Supplementary Material 1

## Data Availability

The dataset supporting the conclusions of this article can be made available upon reasonable request, as permitted by applicable law.
