# Supplementary material for: Nigrostriatal imaging in patients with clinically uncertain Parkinsonian syndromes: a head-to-head comparison between SPECT, PET, and MRI
Source: Eur J Nucl Med Mol Imaging. 2026 Sep 17;53(13):7969–81. doi: 10.1007/s00259-026-08184-8 (PMC13727952; doi:10.1007/s00259-026-08184-8)
Supplement: Supplementary file 1 [file 259_2026_8184_MOESM1_ESM.docx]

**Supplementary Information**

**Nigrostriatal imaging in patients with clinically uncertain parkinsonian syndromes: A head-to-head comparison between SPECT, PET, and MRI**

Gerda Thomsen, BSc^1*^, Kristoffer Brendstrup-Brix, MD^1,2*^, Claus Svarer, MSc, PhD^1^, Ulrich Lindberg, MSc, PhD^3^, Lars H. Pinborg, MD, DMSc^1,2,4^, Karen Kettless, BSc^5^, Ian Law, MD, PhD, DMSc^3^, Steen G. Hasselbalch, MD, DMSc^6^, Bo Biering-Sørensen, MD, MPG^7^, Gitte M. Knudsen, MD, DMSc^1,2^

* shared first authors

1. Neurobiology Research Unit, Department of Neurology, Rigshospitalet, Copenhagen University Hospital, Copenhagen, Denmark
2. Department of Clinical Medicine, Faculty of Health and Medical Sciences, University of Copenhagen, Copenhagen, Denmark
3. Department of Clinical Physiology and Nuclear Medicine, Rigshospitalet, Copenhagen University Hospital, Copenhagen, Denmark
4. Epilepsy Clinic, Department of Neurology, Rigshospitalet, Copenhagen University Hospital, Copenhagen, Denmark
5. Siemens Healthcare A/S, Ballerup, Denmark
6. Danish Dementia Research Centre, Department of Neurology, Rigshospitalet, Copenhagen University Hospital, Copenhagen, Denmark
7. Movement Disorder and Pain Research Center, Department of Neurology, Rigshospitalet, Copenhagen University Hospital, Copenhagen, Denmark

# Supplementary Methods

## MRI acquisition parameters

NM-MRI was acquired with a 3D multi-echo gradient echo sequence with a magnetization transfer contrast (MTC) pulse and the following parameters: TE = 7.5 to 37.5 ms with 7.5 ms increments; TR = 62 ms; flip angle = 30°; field of view (FoV) = 256 × 192 mm; 48 slices with a slice thickness of 1.34 mm; bandwidth = 180 Hz/pixel; in-plane resolution = 0.7 x 0.7 mm; partial Fourier = ⅞; a GRAPPA acceleration factor of 2; and a total scan time of 4 minutes and 31 seconds.

Quantitative data was acquired with two sequential flow compensated bipolar 3D gradient echo sequences with identical imaging parameters at low and high flip angle (6° and 27°) and the following parameters: TR = 29 ms; TE1/ΔTE = 7.5/7.5 ms; GRAPPA acc. = 2 [24 ref lines]; 112 slices per slap; slice oversampling = 7.1%; distance factor = 20%; phase partial Fourier = ⅞; slice partial Fourier = ⅞; bandwidth = 180Hz/px; matrix size = 384 × 197 × 112; voxel resolution = 0.67 × 0.67 × 1.34 mm; FoV = 256 × 192 × 180 mm; and a total scan time of 9 minutes and 36 seconds.

High-resolution, 3D T1-weighted structural MP-RAGE images were acquired with the following parameters: TR = 2000 ms; TE = 2.58 ms; non-selective inverse recovery with TI = 972 ms; flip angle = 8°; FoV = 230 × 230 mm; 224 slices with 0.9 mm slice thickness and a 50% slice distance factor; 0.9 × 0.9 mm isotropic voxels; GRAPPA acceleration by a factor of 2; echo spacing of 7.9 ms; and bandwidth = 170 Hz/Px. For six HCs and five patients, no MP-RAGE was available. Instead, a 3D T1 map from a MP2-RAGE sequence was used, since it had similar contrast. The MP2-RAGE sequence was collected with the following parameters: TR = 4800 ms; TE = 3.35 ms; non-selective inverse recovery with TI = 900 ms and 2500 ms; flip angle = 4° and 5°; FoV = 256 × 224 mm; 224 slices with 1.0 mm slice thickness and a 50% slice distance factor; 1.0 × 1.0 mm isotropic voxels; echo spacing of 6.9 ms; and bandwidth = 240 Hz/Px.

## Preprocessing of MRI

First, images were coregistered in NM-sensitive image acquisition space with affine transforms calculated using *antsRegistration* (ANTs version 3.0.0.0) [1-4]. Since contrast and acquisition volumes differed between STAGE-derived images and NM-MRI, a high-resolution anatomical image was used as an intermediate reference. Transforms from STAGE acquisition space were calculated on a T1 map derived from the reconstruction pipeline, since this image was found to have contrast similar to the anatomical image. All registrations were deemed highly accurate on visual inspection. Brain extraction was then performed on all images of interest using a brain mask calculated on the anatomical image. The brain mask used for brain extraction was derived by segmentation of the anatomical image using SPM12 [5], after which the resulting GM, WM, and CSF probability maps were thresholded at 50% probability, combined, and closed with a mean dilation of non-zero voxels and an erosion, both using a kernel of size 3 × 3 × 3 voxels. Second, NM-sensitive images were corrected for intensity non-uniformity with ANTs’ *N4BiasFieldCorrection* algorithm [6] with weighting relative to WM probability as derived from FSL’s *FAST*. Rescaling was applied at each iteration in order to account for intensity drift. Third, STAGE-derived QSM and NM-sensitive images were median filtered using MATLAB’s *medfilt3* (MATLAB version: 25.1.0.2943329 [R2025a], Natick, Massachusetts: The MathWorks Inc.; 2025). Finally, a combined brainstem and midbrain mask manually drawn on a T1 map from a reference subject (the first HC) was moved to subject NM-sensitive image acquisition space with non-linear registrations using *antsRegistration*. T1 maps were used for these registrations. The resulting brainstem mask was then resliced using SPM12 and applied to QSM and NM-sensitive images.

## Delineation of regions of interest on NM-MRI and QSM

Circular, bilateral reference regions for both NM-MRI and QSM images were drawn in the outermost, anterolateral part of the cerebral crus (**Fig. S1** and **Fig. S2**; avg. NM reference volume = 44.96 [9.5] mm^3^; avg. QSM reference volume = 45.02 [7.8] mm^3^). Mean and standard deviations of signal intensities in the reference regions of the QSM and NM images were calculated and z-score maps were computed by subtracting the mean reference signal from the intensity of each voxel in the brainstem and midbrain mask and dividing by the standard deviation in the reference region.

NM hyperintensities were labeled on NM-sensitive MRI images magnified by a factor of eight for precision. NM ROIs were traced for an average of 4.8 slices (IQR = 4-6) from the most caudal NM signal in the substantia nigra. For each slice, the voxel with the highest signal was selected and the isocontour of a set value closest in space to the selected voxel was drawn. The starting value of the isocontour was defined as the value of the selected voxel minus the difference from the 5^th^ to the 95^th^ percentile of the signal intensities in a square of 10 × 10 voxel size surrounding the initially selected voxel multiplied by a weighting factor. The isocontour was then adjusted by increasing or decreasing the weighting factor in steps of 0.01 (effectively expanding or shrinking the isocontour by 1% of the difference between the 5^th^ to 95^th^ percentile in the region) until the entirety of the NM signal was encapsulated. Since NM hyperintensity in the midbrain is limited to the substantia nigra pars compacta and the small parabrachial pigmented nucleus of the ventral tegmental area [7,8], we take the resulting NM hyperintensity boundaries to be approximate representations of the substantia nigra.

The delineation procedure presented above was repeated for iron-rich hyperintensities on STAGE-derived QSM images. First, the iron-rich red nucleus was manually traced and removed. Iron ROIs were then traced starting from the caudal-most iron signal of the substantia nigra to the cranial-most iron signal at the level just cranial to the red nucleus.

## The sum of ROI z-scores was used as outcome measures for both NM-MRI and iron-sensitive MRI. This measure was chosen since it reflects both the intensity (indirect markers of NM and iron content, respectively) and volume of the ROI. The strategy of using z-score normalized maps instead of raw signal intensities was employed in order to improve observer reproducibility and reduce noise, since voxels with only background signal on average will add up to a z-score of zero and thus not contribute to the sum. These outcome measures are for the remainder of the manuscript referred to as substantia nigra NM and midbrain iron content, respectively.

# References

1.	Avants B, Epstein C, Grossman M, Gee J. Symmetric diffeomorphic image registration with cross-correlation: Evaluating automated labeling of elderly and neurodegenerative brain. Medical Image Analysis. 2008;12: 26–41. doi:10.1016/j.media.2007.06.004

2.	Klein A, Andersson J, Ardekani BA, Ashburner J, Avants B, Chiang M-C, et al. Evaluation of 14 nonlinear deformation algorithms applied to human brain MRI registration. NeuroImage. 2009;46: 786–802. doi:10.1016/j.neuroimage.2008.12.037

3.	Murphy K, Van Ginneken B, Reinhardt JM, Kabus S, Ding K, Deng X, et al. Evaluation of Registration Methods on Thoracic CT: The EMPIRE10 Challenge. IEEE Trans Med Imaging. 2011;30: 1901–1920. doi:10.1109/TMI.2011.2158349

4.	Avants BB, Tustison NJ, Song G, Cook PA, Klein A, Gee JC. A reproducible evaluation of ANTs similarity metric performance in brain image registration. Neuroimage. 2011;54: 2033–2044. doi:10.1016/j.neuroimage.2010.09.025

5.	Penny WD, Friston KJ, Ashburner JT, Kiebel SJ, Nichols TE. Statistical Parametric Mapping: The Analysis of Functional Brain Images. Elsevier Science; 2011. Available: https://books.google.dk/books?id=G_qdEsDlkp0C

6.	Tustison NJ, Avants BB, Cook PA, Yuanjie Zheng, Egan A, Yushkevich PA, et al. N4ITK: Improved N3 Bias Correction. IEEE Trans Med Imaging. 2010;29: 1310–1320. doi:10.1109/TMI.2010.2046908

7.	He N, Chen Y, LeWitt PA, Yan F, Haacke EM. Application of Neuromelanin MR Imaging in Parkinson Disease. J Magn Reson Imaging. 2023;57: 337–352. doi:10.1002/jmri.28414

8.	He N, Ghassaban K, Huang P, Jokar M, Wang Y, Cheng Z, et al. Imaging iron and neuromelanin simultaneously using a single 3D gradient echo magnetization transfer sequence: Combining neuromelanin, iron and the nigrosome-1 sign as complementary imaging biomarkers in early stage Parkinson’s disease. NeuroImage. 2021;230: 117810. doi:10.1016/j.neuroimage.2021.117810

**Figure S1. Observer reproducibility of delineation of brain region for determination of neuromelanin on MRI.** **A, B:** Exemplary region of interest (ROI) delineations in a healthy control (**A**) and a Parkinson’s disease patient (**B**). First and second repetitions of ROI delineations are outlined in yellow and red, respectively. Bilateral reference regions in the outermost, anterolateral part of the cerebral crus are outlined in white. **C, D:** Correlation plot (**C**) and Bland-Altman plot (**D**) of the resulting sum of z-scores on first and second ROI delineation repetitions. Both repetitions were performed by the same rater. Classification defines the clinical evaluation based on visual inspection and DAT SBR values. No DAT images for classification were available for healthy controls.

**Figure S2. Observer reproducibility of delineation of brain region for determination of iron on MRI.** **A, B:** Exemplary region of interest (ROI) delineations in a healthy control (**A**) and a Parkinson’s disease patient (**B**). First and second repetitions of ROI delineations are outlined in yellow and red, respectively. Bilateral reference regions in the outermost, anterolateral part of the cerebral crus are outlined in white. **C, D:** Correlation plot (**C**) and Bland-Altman plot (**D**) of the resulting sum of z-scores on first and second ROI delineation repetitions. Both repetitions were performed by the same rater. Classification defines the clinical evaluation based on visual inspection and DAT SBR values. No DAT images for classification were available for healthy controls.

**Figure S3. Standardized neuromelanin and iron hyperintensities relative to age and intracranial volume.** Effects of age (**A, C**) and intracranial volume (**B. D**) on substantia nigra neuromelanin (**A, B**) and QSM (**C, D**) hyperintensity. A linear regression model was fitted to data from the healthy controls only and used for subsequent adjustment for age and intracranial volume in both healthy controls and patients. Patient data are shown here for reference only.

# Table S1

|  | **SPECT Mean [SD]** | **PET Mean [SD]** | **Cohen’s *d* [95% CI]** | ***p_FWER_*** |
| --- | --- | --- | --- | --- |
| **Waiting time from injection to scan** | 3.70 [0.87] | 3.56 [0.93] | 0.12 [-0.26, 0.50] | 0.54 |
| **Injection of tracer** | 4.44 [0.64] | 4.22 [0.89] | 0.30 [-0.09, 0.68] | 0.27 |
| **Scanning procedure** | 4.19 [0.83] | 4.33 [0.62] | -0.22 [-0.60, 0.16] | 0.34 |
| **General experience** | 4.78 [0.42] | 4.33 [0.78] | 0.59 [0.18, 1.00] | 0.02 |

**Table S1. Patient reports on satisfaction with and comfort during DAT imaging for PET and SPECT.** Patients were asked to rate each item on a scale from one to five with five representing the highest satisfaction or comfort. Differences between SPECT and PET scan session reports were tested with paired, two-tailed Student’s t-tests, and p-values were corrected for multiple comparisons with the Benjamini-Hochberg method (four tests).

**Figure S4. Correlations between putamen and caudate asymmetry indices from [^18^F]FE-PE2I** **PET and [^123^I]FP-CIT SPECT and corresponding receiver operating characteristics (ROC) curves.** **A, B:** Correlation between PET and SPECT asymmetry indices in putamen and caudate nucleus. Data points are colored according to the clinical evaluation (based on visual inspection and DAT SBR values) with normal scans colored in yellow and abnormal scans in orange. **C:** ROC curves for putamen and caudate nucleus asymmetry indices as evaluated on SPECT (blue colors), and PET (orange and yellow colors) images. Areas under the curve (AUC) for each ROC curve are displayed under the diagonal.

**Figure S5. Bland-Altman plots between specific binding ratios (SBR) and related quantitative measures from** **[^18^F]FE-PE2I** **PET and [^123^I]FP-CIT SPECT.** Classification defines the clinical evaluation based on visual inspection and DAT SBR values. **A, B, C:** SBR of PET relative to SBR of SPECT in the striatum, caudate nucleus, and putamen. **D:** Relation between putamen and caudate nucleus SBR as measured with PET relative to SPECT. **E:** Striatal asymmetry index (SAI) as evaluated on PET relative to SPECT.

**Figure S6. Standardized neuromelanin hyperintensities across diagnostic groups.** **A:** Adjusted sum of neuromelanin (NM) z-scores in the region of interest (ROI) per diagnosis group. The central point on the error bar represents the group mean with whiskers extending one standard deviation in each direction. **B:** Group differences between Parkinson’s disease (PD) and PD+ patients, patients with other neurological diseases (iNPH, VP, MCI, and tremor), and individuals without neurological disease. **C:** Group differences based on clinical evaluation of DAT images, which included visual inspection and evaluation of SBR values. Group differences were tested with unpaired, two-sided Student’s t-tests, and p-values were corrected for multiple comparisons with the Benjamini-Hochberg method (three tests). Cohen’s *d* values are displayed with corresponding 95% confidence intervals in parentheses. PD, Parkinson’s disease; PD+, Parkinson’s disease plus (progressive supranuclear palsy, PD with dementia, Lewy body dementia, non-specific basal ganglia neurodegenerative disease); VP, vascular pseudoparkinsonism; DIP, drug-induced parkinsonism; iNPH, idiopathic normal pressure hydrocephalus; MCI, mild cognitive impairment; HC, healthy control.

**Figure S7. Standardized QSM hyperintensities across diagnostic groups.** **A:** Adjusted sum of QSM z-scores in the region of interest (ROI) per diagnosis group. The central point on the error bar represents the group mean with whiskers extending one standard deviation in each direction. **B:** Group differences between Parkinson’s disease (PD) and PD+ patients, patients with other neurological diseases (iNPH, VP, MCI, and tremor), and individuals without neurological disease. **C:** Group differences based on clinical evaluation of DAT images, which included visual inspection and evaluation of SBR values. Group differences were tested with unpaired, two-sided Student’s t-tests, and p-values were corrected for multiple comparisons with the Benjamini-Hochberg method (three tests). Cohen’s *d* values are displayed with corresponding 95% confidence intervals in parentheses. PD, Parkinson’s disease; PD+, Parkinson’s disease plus (progressive supranuclear palsy, PD with dementia, Lewy body dementia, non-specific basal ganglia neurodegenerative disease); VP, vascular pseudoparkinsonism; DIP, drug-induced parkinsonism; iNPH, idiopathic normal pressure hydrocephalus; MCI, mild cognitive impairment; HC, healthy control.

# Figure S8. Correlations between standardized neuromelanin hyperintensities and duration of symptoms in Parkinson's disease patients. Datapoints are colored according to Hoehn & Yahr stage. Correlation was tested with a Pearson correlation test, and 95% confidence intervals on the correlation coefficient are displayed in parentheses. Unadjusted p-value.
